# Applications of digital health for public health responses to COVID-19: a systematic scoping review of artificial intelligence, telehealth and related technologies

**DOI:** 10.1038/s41746-021-00412-9

**Published:** 2021-02-26

**Authors:** Dinesh Visva Gunasekeran, Rachel Marjorie Wei Wen Tseng, Yih-Chung Tham, Tien Yin Wong

**Affiliations:** 1 Singapore Eye Research Institute (SERI), Singapore National Eye Center (SNEC), Singapore, Singapore; 2 Yong Loo Lin School of Medicine, National University of Singapore (NUS), Singapore, Singapore; 3 Duke-NUS Medical School, Singapore, Singapore

**Keywords:** Infectious diseases, Public health

## Abstract

The coronavirus disease 2019 (COVID-19) pandemic has overwhelmed healthcare services, faced with the twin challenges in acutely meeting the medical needs of patients with COVID-19 while continuing essential services for non-COVID-19 illnesses. The need to re-invent, re-organize and transform healthcare and co-ordinate clinical services at a population level is urgent as countries that controlled initial outbreaks start to experience resurgences. A wide range of digital health solutions have been proposed, although the extent of successful real-world applications of these technologies is unclear. This study aims to review applications of artificial intelligence (AI), telehealth, and other relevant digital health solutions for public health responses in the healthcare operating environment amidst the COVID-19 pandemic. A systematic scoping review was performed to identify potentially relevant reports. Key findings include a large body of evidence for various clinical and operational applications of telehealth (40.1%, *n* = 99/247). Although a large quantity of reports investigated applications of artificial intelligence (AI) (44.9%, *n* = 111/247) and big data analytics (36.0%, *n* = 89/247), weaknesses in study design limit generalizability and translation, highlighting the need for more pragmatic real-world investigations. There were also few descriptions of applications for the internet of things (IoT) (2.0%, *n* = 5/247), digital platforms for communication (DC) (10.9%, 27/247), digital solutions for data management (DM) (1.6%, *n* = 4/247), and digital structural screening (DS) (8.9%, *n* = 22/247); representing gaps and opportunities for digital public health. Finally, the performance of digital health technology for operational applications related to population surveillance and points of entry have not been adequately evaluated.

## Introduction

The coronavirus disease 2019 (COVID-19) pandemic has crippled both economies and health systems, killing more than 1 million people, with threats of resurgence even as many nations control initial outbreaks^1,2^. Many health systems are overwhelmed^3,4^, with this trend being more pronounced in front-line emergency services and mental health services^5–7^. Conservative modelling has indicated that certain health systems are particularly vulnerable, including many developing countries in Asia with limited healthcare capacity, along with shortages of beds in hospitals and intensive care units (ICUs) in African countries^8,9^. Health systems need to rapidly re-organize resources and restructure clinical services at a population level to minimize the risk of healthcare-associated transmission, as well as meet public health requirements for continued surveillance, risk mitigation, and containment^2,4^.

Digital health technologies, such as telehealth, artificial intelligence (AI) and big data predictive analytics, offer substantial promise to mitigate the effects of COVID-19 by enhancing population-level public health responses. Some of these digital solutions have already been piloted and deployed to address the challenges of COVID-19^10,11^. However, while there have been exciting isolated reports of real-world development and validation of these digital solutions, recent literature has also highlighted significant challenges in deployment and scale-up, and limitations of clinical trials that are of varied quality and design^12,13^. Therefore, it is presently unclear what digital health solutions, if any, have been successfully deployed and applied in the public health responses to the COVID-19 pandemic.

This manuscript is a systemic review of digital health applications for population-level public health responses during the first 6 months of the pandemic. We used a scoping review approach to map out the range and nature of evidence, in order to answer our fundamental question: “What forms of digital health had been applied for public health responses to COVID-19?”.

## Results

We retrieved an initial 1904 unique records by the search. All titles and abstract information available in the database were reviewed during screening, and 1559 reports were excluded. 345 full-text reports were then assessed for eligibility. The 345 reports identified on screening originated from over 15 countries and regions (Supplemental Fig. 1). After full-text articles were assessed for eligibility, 247 reports were included in this scoping review for data charting and analysis (Screening flow diagram in Supplemental Fig. 2). The study design and other key features of the included articles are described in Table 1. Only 20 articles (8.1%) investigated patient and/or provider acceptance of these technologies, whereby 17 studies focused primarily on acceptance while three cross-sectional studies had included assessment of acceptance. The technology domains that were most frequently described for responses to COVID-19 were AI (44.9%, *n* = 111/247), telehealth (40.1%, *n* = 99/247), and big data (36.0%, *n* = 89/247).

**Table 1:** Details of reports included in this study.

Country of origin	Single country	176 (71.3%)
	Multiple countries/Big data sets	71 (28.7%)
Type of report	Published research	212 (85.8%)
	Pre-print (bioRxiv, medRxiv, preprints.org, etc.)	35 (14.2%)
Study design	Randomised controlled trials (RCTs)	0
	Cohort study	52 (21.1%)
	Case–control study	2 (1.6%)
	Cross-sectional study/case series	44 (17.8%)
	Survey on patient/Provider acceptance	17 (6.9%)
	Case report of a patient	6 (2.4%)
	Description of a technology solution	126 (51.0%)
Method of analysis	Prospective intention to treat (ITT) analysis	3 (1.2%)
	Prospective non-ITT analysis	28 (11.3%)
	Retrospective analysis	51 (20.6%)
	Descriptive analysis (non-interventional)	165 (66.8%)
Technology domain	Artificial intelligence (AI)	111 (44.9%)
	Big data	89 (36.0%)
	Internet of things (IoT)	5 (2.0%)
	Telehealth (including mHealth apps and web-based solutions)	99 (40.1%)
	Digital platforms for communication (DC)	27 (10.9%)
	Digital solutions for data management (automated data normalisation, blockchain, etc.)	4 (1.6%)
	Digital structural screening for COVID-19 therapies (DS)	22 (8.9%)
Disease topic	Possible COVID-19	143 (57.9%)
	Non-COVID-19 illness	106 (42.9%)
	Either/Both the above groups	58 (23.5%)

**Fig. 1:**
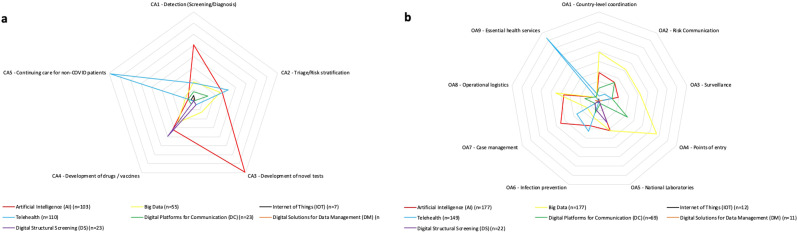
Public health applications of digital health described in COVID-19. Spider diagram of (**a**) clinical applications and (**b**) operational applications for the digital health technology domains described in COVID-19. Scale for the radial axes of this chart are standardized at 10 units per layer.

The complete spectrum of CAs and OAs of these digital health technology domains in the context of COVID-19 are detailed in Table 2. These are further visualized in the form of a Spider diagrams (Fig. 1) and detailed matrix cross-tabular table (Supplemental Fig. 3) to map the evidence for these digital health solutions, indicating the percentage of reports that have topical coverage of each clinical and/or operational application within each technology domain.

**Table 2:** Clinical and operational applications of digital health technologies in COVID-19.

Application	Description	Primary data	Narrative discussion	Total topical coverage
Clinical applications (CA)	Detection (screening/diagnosis)	29 (11.7%)	37 (15.0%)	66 (26.7%)
	Triage/Risk stratification (predicting mortality, severity, duration of admission, etc.)	19 (7.7%)	26 (10.5%)	45 (18.2%)
	Developing treatment (novel or repurposed drugs or vaccines)	13 (5.3%)	10 (4.0%)	23 (9.3%)
	Developing novel tests	4 (1.6%)	18 (7.3%)	22 (8.9%)
	Continuing care for non-COVID patients	33 (13.4%)	49 (19.8%)	82 (33.2%)
Operational applications (OA) prioritised for country-level responses by the World Health Organisation (WHO)	Country-level coordination, planning and monitoring (CPM)	30 (12.1%)	31 (12.6%)	61 (24.7%)
	Communication—risk communication and community engagement	18 (7.3%)	23 (9.3%)	41 (16.6%)
	Surveillance, rapid response teams, and case investigations (e.g. contact tracing)	9 (3.6%)	8 (3.2%)	17 (6.9%)
	Points of entry	1 (0.4%)	1 (0.4%)	2 (0.8%)
	National laboratories	3 (1.2%)	10 (4.0%)	13 (5.3%)
	Infection prevention and control	7 (2.8%)	41 (16.6%)	48 (19.4%)
	Case management	37 (15.0%)	71 (28.7%)	108 (43.7%)
	Operational support and logistics	16 (6.5%)	34 (13.8%)	50 (20.2%)
	Maintaining essential health services	29 (11.7%)	51 (20.6%)	80 (32.4%)

Despite a large number of reports describing promising AI and big data applications in the pandemic^13^, we found that minimal investigations for patient and/or provider acceptance have been reported. This is despite numerous reports of these tools being widely applied for surveillance or interpretation of chest imaging scans for operational efficiencies in overloaded healthcare services. Furthermore, few investigations of IoT solutions have been reported despite a large number of these solutions being deployed at the population level for the monitoring of high risk patients under quarantine, such as returning travelers or contacts of confirmed cases. That said, there have been a surprisingly large number of reports regarding applications of DCs such as digital messaging communications platforms, national/organizational websites for information dissemination (1-way), or online health communities (OHCs) that facilitate discussions (2-way). These categories of reports are further detailed in Table 3 using the report assessment criteria defined in the methodology section (screening reports).

**Table 3:** Characterisation of the evidence for digital health technologies from included reports.

		AI (*n* = 111)	Big data (*n* = 89)	IoT (*n* = 5)	Telehealth (*n* = 99)	DC (*n* = 27)	DM (*n* = 4)	DS (*n* = 22)
Translational relevance (Method of analysis)	Prospective intention to treat (ITT) analysis	0	0	0	3 (3.0%)	1 (3.7%)	0	0
	Prospective non-ITT analysis	3 (2.7%)	6 (6.7%)	1 (20.0%)	22 (22.2%)	4 (14.8%)	0	0
	Retrospective analysis	7 (6.3%)	12 (13.5%)	0	35 (35.4%)	2 (7.4%)	0	0
	Descriptive analysis (non-interventional)	101 (91.0%)	71 (79.8%)	4 (80.0%)	39 (39.4%)	20 (74.1%)	4 (100%)	22 (100%)
Strength of evidence (Study design)	Randomised controlled trials (RCTs)	0	0	0	0	0	0	0
	Cohort study	5 (4.5%)	9 (10.1%)	0	41 (41.4%)	3 (11.1%)	0	0
	Case–control study	1 (0.9%)	1 (1.1%)	0	1 (1.0%)	0	0	0
	Cross-sectional/ case series	13 (11.7%)	14 (15.7%)	1 (20.0%)	20 (20.2%)	3 (11.1%)	0	0
	Survey on patient/Provider acceptance	0	1 (1.1%)	0	16 (16.2%)	4 (14.8%)	0	0
	Case report of a patient	1 (0.9%)	0	0	6 (6.1%)	0	0	0
	Description of a technology solution	91 (82.0%)	64 (71.9%)	4 (80.0%)	15 (15.2%)	17 (63.0%)	4 (100.0%)	22 (100%)

**Fig. 2:**
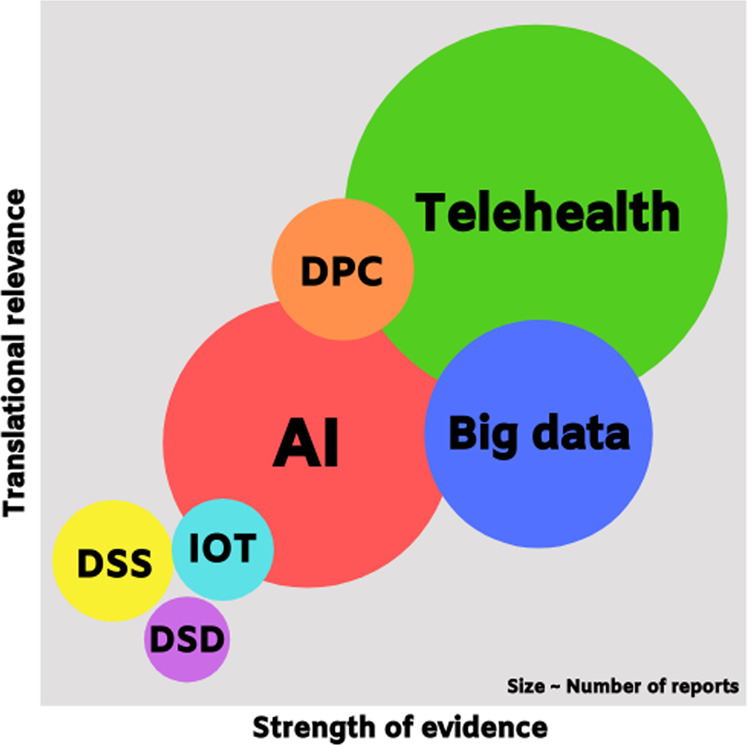
Bubble plot of translational relevance and strength of evidence for included reports. The scales for “Translational relevance” and “Strength of evidence” are applied based on study design, participant recruitment and follow-up as described in the Methodology section.

There was a paucity of reports describing the performance of digital health technologies applied at points of entry and national laboratories. The varying quality of study design and methods of analyses are further depicted in the form of a bubble plot in Fig. 2 using the same report assessment criteria, highlighting the shortage of investigations for DM, IoT, DS and DC, as well as methodological limitations despite the large number of reported studies for AI and big data applications. Notably, there was a surprising prominence of reports about DCs (Fig. 2) for triage^14–16^, co-ordination^16–18^, and public health communication addressing misinformation, resource availability, and evolving guidelines^19–22^.

## Discussion

Our systematic scoping review provides an overview of digital health technology that were used for clinical and/or operational applications for population-level public health responses in the first 6 months of the COVID-19 pandemic. Our findings build on recent editorial and perspective articles that provide a subjective narrative overview of various digital health topics that could be potentially applied in public health responses to COVID-19^10,11,23^. The systematic and pragmatic approach of this scoping review provides a map of existing reports at this critical juncture of the pandemic as countries develop population health strategies for safe re-opening. We believe that this serves as a crucial reference for public healthcare systems regarding potential impact and relevance of different digital technologies to prioritise resources and efforts to address the challenges presented by COVID-19. In addition, we highlight significant gaps in the literature that can be addressed through the conduct of research concurrently with the deployment of these solutions.

The need for rapid adoption of digital health technology has been suggested and driven by the unprecedented scale in the impact of the COVID-19 pandemic due to an increasingly connected global ecosystem with mass travel, urban overcrowding, and information from social and digital media^11^. These factors did not feature as prominently in previous major infectious disease outbreaks such as severe acute respiratory distress syndrome (SARS) and middle east respiratory syndrome (MERS)^24,25^. In particular, the deluge of misinformation during this pandemic has drowned out official information, in what has been dubbed an “infodemic” by WHO^26^. Coupled with evolving recommendations as scientists gradually uncover more information about this virus^23^, the infodemic has needlessly fueled growing paranoia and anxiety among the public^7^, as well as confusion for patients with chronic diseases who seek to continue the care for their medical problems^27^. In this regard, DC is at the forefront to address the infodemic and provide transparent information and updates, as reflected in the prominence of relevant reports (Fig. 2).

While there are a range of digital health technology and the maturity of some (e.g., AI) has paved the way for the digitization of clinical and operational responses to contain the pandemic^28^, there remain significant challenges and gaps in adoption, scale-up and integration into healthcare systems, even in developed countries^29^. For example, there continues to be ethical concerns with population-level deployment of these tools, particularly in the case of surveillance technologies without individual consent, presenting new ethical and privacy concerns that need to be addressed^30,31^. Moreover, although vulnerable regions with limited health system capacity are likely to benefit the most from scalable digital tools^32^, many have barriers to technology implementation illustrated in earlier technology reports^33^. These regions will require concerted support and public health coordination for the year ahead^23^, at least until a safe and effective vaccine or treatment is readily available. Without this, limiting the human toll and addressing infectious reservoirs will remain a formidable challenge, potentially crippling these fragile health systems.

The scoping review approach provided for a detailed account of the spectrum of relevant literature at this critical juncture of the pandemic. Gaps in the literature that have been identified in this review include assessments of digital health technologies for operational applications at points of entry and for population surveillance (Fig. 1). Furthermore, although there was a large quantity of reports investigating applications of AI and big data, limitations in study design curb generalizability and translation. These results indicate that there is a pressing need for more investigations of IoT, DC, DM, and DS digital health technologies, as well as underscore the need for better quality studies of digital health such as AI and big data applications using prospective, pragmatic study designs (Fig. 2)^34^.

The strengths of this review include its timeliness in the context of the ongoing pandemic, systematic article inclusion and data extraction, as well as the scoping review approach for an in-depth analysis of the literature. Added benefits of this review include an a priori protocol and involvement of stakeholders with relevant experience developing digital health and deployment in clinical services before and during COVID-19^35^. We have specifically included in this review only digital technologies with applications for population-level public health responses during COVID-19. Our search strategy was limited to reports that self-identified with relevant search terms (Supplementary note 1) selected to improve the yield of reports about digital technologies with relevance to population-level public health responses to COVID-19 given the timeliness of this topic We have not included other digital technologies such as those regarding fitness trackers, augmented reality (AR) or virtual reality (VR) digital health tools^29^.

The limited number of studies investigating patient and provider acceptance of these tools (<10% of reports) also highlights the need for greater participatory research involving stakeholders to increase the likelihood of sustained adoption beyond the pandemic^26,36^. This is advocated on the basis of a growing body of evidence surrounding the complexity of digital health solutions due to their interactions with operational and interpersonal aspects of clinical care beyond the target condition(s)^30,36–38^. Therefore, digital health solutions may need to be evaluated as clinical care pathway interventions rather than isolated tools, in order to achieve holistic assessment and inform successful implementation^39^.

In conclusion, our study provides a rapid scoping review of digital health applications described in the first six months of the pandemic, highlighting potential applications and gaps in the literature for the consideration of clinicians, administrators, and researchers. More studies investigating specific applications of digital health to develop relevant scalable public health responses are highlighted, in particular, the pressing need for researchers to formally evaluate digital health applications for population surveillance and points of entry. Finally, there is a general need for better methodological design in the investigation of digital health applications prospectively using pragmatic approaches to better inform public health responses. The use of participatory approaches in the deployment and assessment of these tools will also yield crucial insights to enable sustained adoption during and beyond the pandemic.

## Methods

We conducted a systematic review in accordance with the preferred reporting items for systematic reviews and meta-analyses (PRISMA) guidelines extension for scoping reviews (PRISMA-ScR). The review was pre-registered in open science framework (OSF, registration number: osf.io/8nbgj). To be included in the review, papers needed to provide original descriptions of clinical and/or operational applications of digital health technology or solutions in the context of COVID-19 for population-level public health responses. All English-language peer-reviewed reports and pre-prints published within the first 6 months of the pandemic are included. Pre-prints are included due to the extremely current nature of this topic. The completed PRISMA-ScR checklist is included (Supplemental Table 1).

### Search strategy and selection criteria

To identify potentially relevant reports, databases were searched from the time of the initial announcement from WHO regarding a cluster of cases of pneumonia in Wuhan on 31 Dec 2019^40^, to 1 July 2020. The search was conducted on 2 July 2020 and exported to Microsoft excel for screening and charting. Electronic bibliographic databases of published research in Pubmed including MEDLINE, IEE explore, and databases for research pre-prints including medrXiv (health sciences), arXiv (engineering), and bioRxiv (biology), given the cross-disciplinary nature of the search topic involving both health sciences and information systems. The search strategies were drafted and refined through study team discussion. Search terms selected for the literature search include the digital health technology domains and the target application context of the pandemic using Boolean operators (OR/AND). The final detailed search strategy for Pubmed is included in this publication (Supplementary note 1).

Randomized-controlled trials, cohort studies, case–control studies, case series, descriptions of technology solutions or case reports of digital health technology and solutions for clinical and/or operational applications in COVID-19 are included. Reports were excluded if they did not fit into the conceptual framework of clinical and/or operational applications for public health responses applied to this study, such as descriptions of digital health for residency training or continuing medical education. Editorials, perspective articles, narrative or other reviews without original data, and study protocols are also excluded.

### Screening reports

Study selection was determined by review of available information from study title and abstract in the indexed database for relevance to digital health clinical and/or operational applications for public health responses in the context of COVID-19. Data charting was completed based on all accessible information in the study manuscript. A standardized study screening manual, including a data charting form along with an explanation and elaboration document in the form of a coding manual (Supplementary note 2) was developed by the study team by group consensus.

The report quality assessment criteria used in this study were extrapolated from distillation of the oxford center for evidence-based medicine (OCEBM) construct^41^, to facilitate greater granularity and relevance to translation for this review. This was done with an aim to provide practical information for decision makers to inform ongoing responses to the pandemic and identify gaps in the literature for researchers looking to evaluate ongoing applications of digital health technologies. Studies are thereby categorized based on the strength of evidence, ranging from case reports to the ideal randomized-controlled trial (RCT) methodology, as well as the translational relevance depending whether prospective or retrospective data was used, and whether an intention-to-treat approach to evaluate the technology “as offered” was adopted to reduce bias and missing data^39,42,43^.

The coding manual (Supplementary note 2) details how digital health technology or solutions described in these reports were characterized based on technology domains^10^, including artificial intelligence (AI), big data analytics, internet of things (IoT), telehealth, digital platforms for communication (DC), digital solutions for data management (DM), and digital structural screening (DS). The coding of clinical applications (CAs) were indicated based on clinical priorities for patients with COVID-19 such as detection, triage, developing tests/treatment, as well as continuing care for patients with non-COVID ailments^44,45^. Finally, the coding of potential relevance to operational applications (OAs) were indicated based on descriptions of the 9-pillars of country-level public health responses as recommended by the WHO^46^.

### Data charting and analysis

To increase consistency of study screening among reviewers, reviewer 1 (DG) piloted the study screening manual for database search and study selection based on title/abstract information available in the databases. Subsequently, reviewer 2 (RT) independently cross-checked study selection for 10% of all articles identified in the database search, using a computer-generated random sequence (www.randomizer.org). Both reviewers then discussed results, and amended the screening manual before the data charting step.

Subsequently, both reviewer 1 and 2 independently piloted the study screening manual for evaluating the eligibility of 10% of all identified full-text reports using a computer-generated random sequence (www.randomizer.org), along with complete data charting for the included articles. Both reviewers then discussed results and amended the screening manual. Finally, reviewers 1 and 2 independently completed assessment of the remaining full-text reports for eligibility along with data charting for all included reports.

Any disagreements on study selection and data charting during pilot testing were resolved by consensus, or otherwise tie-breaker by reviewer 3 (YT) if needed. Where interrater agreement was low (Cohen’s kappa coefficient < 0.8), a repeat sampling of 10% of all relevant reports was conducted with disagreements resolved by consensus. Data from the coding of included studies were analysed quantitatively whereby missing data were handled by pairwise deletion without imputation. We grouped studies by technology domains and summarized the clinical and operational applications described to identify key trends in the literature and knowledge gaps for future research. All findings are synthesized using a narrative review approach. Key results are summarized using Spider diagrams, matrix cross-tabular table of the published clinical/operational applications as well as a bubble plot of the reports depicting the strength of evidence and translational relevance for each technology domain.

### Reporting summary

Further information on research design is available in the Nature Research Reporting Summary linked to this article.

## Supplementary information

Supplementary Information

Reporting Summary

## Data Availability

All included reports from which data was generated and/or analysed in this systematic review are included in the published article and Supplemental Information.
