# Supplementary material for: Applications of digital health for public health responses to COVID-19: a systematic scoping review of artificial intelligence, telehealth and related technologies
Source: NPJ Digit Med. 2021 Feb 26;4:40. doi: 10.1038/s41746-021-00412-9 (PMC7910557; doi:10.1038/s41746-021-00412-9)
Supplement: Supplementary file 2 — Reporting Summary [file 41746_2021_412_MOESM2_ESM.pdf]

nature research

Corresponding author(s): Professor Tien Y Wong

Last updated by author(s): Dec 2, 2020

Reporting Summary

Nature Research wishes to improve the reproducibility of the work that we publish. This form provides structure for consistency and transparency in reporting. For further information on Nature Research policies, see our Editorial Policies and the Editorial Policy Checklist.

Statistics

For all statistical analyses, confirm that the following items are present in the figure legend, table legend, main text, or Methods section.

n/a	Confirmed	
☐	☒	The exact sample size (n) for each experimental group/condition, given as a discrete number and unit of measurement
☒	☐	A statement on whether measurements were taken from distinct samples or whether the same sample was measured repeatedly
☒	☐	The statistical test(s) used AND whether they are one- or two-sided<br>*Only common tests should be described solely by name; describe more complex techniques in the Methods section.*
☒	☐	A description of all covariates tested
☒	☐	A description of any assumptions or corrections, such as tests of normality and adjustment for multiple comparisons
☒	☐	A full description of the statistical parameters including central tendency (e.g. means) or other basic estimates (e.g. regression coefficient) AND variation (e.g. standard deviation) or associated estimates of uncertainty (e.g. confidence intervals)
☒	☐	For null hypothesis testing, the test statistic (e.g. F, t, r) with confidence intervals, effect sizes, degrees of freedom and P value noted<br>*Give P values as exact values whenever suitable.*
☒	☐	For Bayesian analysis, information on the choice of priors and Markov chain Monte Carlo settings
☒	☐	For hierarchical and complex designs, identification of the appropriate level for tests and full reporting of outcomes
☒	☐	Estimates of effect sizes (e.g. Cohen's d, Pearson's r), indicating how they were calculated

Our web collection on statistics for biologists contains articles on many of the points above.

Software and code

Policy information about availability of computer code

Data collection	NA
Data analysis	NA

For manuscripts utilizing custom algorithms or software that are central to the research but not yet described in published literature, software must be made available to editors and reviewers. We strongly encourage code deposition in a community repository (e.g. GitHub). See the Nature Research guidelines for submitting code & software for further information.

Data

Policy information about availability of data

All manuscripts must include a data availability statement. This statement should provide the following information, where applicable:

- Accession codes, unique identifiers, or web links for publicly available datasets
- A list of figures that have associated raw data
- A description of any restrictions on data availability

Uploaded in Appendix 4

Field-specific reporting

Please select the one below that is the best fit for your research. If you are not sure, read the appropriate sections before making your selection.

☒ Life sciences ☐ Behavioural & social sciences ☐ Ecological, evolutionary & environmental sciences

For a reference copy of the document with all sections, see nature.com/documents/nr-reporting-summary-flat.pdf

Life sciences study design

All studies must disclose on these points even when the disclosure is negative.

Sample size	For this systematic scoping review, databases were searched from the time of the initial announcement from WHO regarding a cluster of cases of pneumonia in Wuhan on 31 Dec 2019, 14 to 1 July 2020. The search was conducted on 2 July 2020 and exported to Microsoft excel for screening and charting. Electronic bibliographic databases of published research in Pubmed including MEDLINE, IEE explore, and databases for research pre-prints including medrXiv (health sciences), arXiv (engineering), and bioRxiv (biology), given the cross-disciplinary nature of the search topic involving both health sciences and information systems. The search strategies were drafted and refined through study team discussion. Search terms selected for the literature search include the digital health technology domains and the target application context of the pandemic using Boolean operators (OR/AND). The final detailed search strategy for Pubmed is included in this publication (Appendix 2).
Data exclusions	Reports were excluded if they did not fit into the conceptual framework of clinical and/or operational applications for public health responses applied this study, such as descriptions of digital health for residency training or continuing medical education. Editorials, perspective articles, narrative or other reviews without original data, and study protocols are also excluded.
Replication	We conducted a systematic review in accordance with the Preferred Reporting Items for Systematic reviews and Meta-Analyses (PRISMA) guidelines extension for scoping reviews (PRISMA-ScR). The review was pre-registered in Open Science Framework (OSF, registration number: osf.io/8nbgj). To be included in the review, papers needed to provide original descriptions of clinical and/or operational applications of digital health technology or solutions in the context of COVID-19 for population-level public health responses. All English-language peer-reviewed reports and pre-prints published within the first 6 months of the pandemic are included. Pre-prints are included due to the extremely current nature of this topic. The completed PRISMA-ScR checklist is included (Appendix 1).
Randomization	NA
Blinding	NA

Reporting for specific materials, systems and methods

We require information from authors about some types of materials, experimental systems and methods used in many studies. Here, indicate whether each material, system or method listed is relevant to your study. If you are not sure if a list item applies to your research, read the appropriate section before selecting a response.

Materials & experimental systems

n/a	Involved in the study	
☒	☐	Antibodies
☒	☐	Eukaryotic cell lines
☒	☐	Palaeontology and archaeology
☒	☐	Animals and other organisms
☒	☐	Human research participants
☒	☐	Clinical data
☒	☐	Dual use research of concern

Methods

n/a	Involved in the study	
☒	☐	ChIP-seq
☒	☐	Flow cytometry
☒	☐	MRI-based neuroimaging
